# Lethal Neural Tube Defects: Reports of Anencephaly and Craniorachischisis Cases and Literature Review

**DOI:** 10.1155/2023/4017625

**Published:** 2023-12-19

**Authors:** Alemayehu Shiferaw Lema, Jemila Salih Suleyman

**Affiliations:** Department of Forensic Medicine and Toxicology, St. Paul's Hospital Millennium Medical College, Addis Ababa, Ethiopia

## Abstract

Neural tube defects are serious birth defects of the central nervous system that result from a multifaceted disruption of normal embryogenesis of the nervous system. Although largely preventable, they nonetheless pose a serious threat to global morbidity, disability, mortality, and financial expenses. Despite this, it has been neglected and has only been the subject of limited research until recently. Furthermore, surveillance efforts for neural tube defects remain limited, and no decline in defects has been documented in less developed countries. Here, we report two cases of craniorachischisis and one case of discordant twins for anencephaly. Moreover, the relevant works of literature that are necessary to understand and address this unrelenting phenomenon are provided.

## 1. Introduction

Globally, congenital abnormalities play an important role in perinatal and infant morbidity and mortality [1, 2]. As significant congenital malformations of the nervous system, neural tube defects result from a multifaceted disruption of normal embryogenesis of the nervous system. Although largely preventable, they nonetheless pose a serious threat to global morbidity, disability, mortality, and financial expenses [1, 2]. According to estimates of the global and regional prevalence of neural tube defects (NTDs) for 2015, about 260,100 (95% CI: 213,800–322,000) newborns around the world are born annually with neural tube defects excluding early spontaneous fetal losses [3]. In 2015, there were an estimated 117,100 NTD-affected pregnancies that ended in stillbirth or elective terminations of pregnancy for fetal anomalies. Besides, of NTD-affected live births, about 117,900 resulted in under-5 deaths [3]. Other similar estimates showed that the disability-adjusted life years from NTDs exceed 8.6 million [4].

The burden of neural tube defects shows a discrepancy between geographic locations worldwide [2, 3]. According to estimates of the global and regional prevalence of neural tube defects for 2015, the frequency of NTDs per 10,000 births was 7.5 in North America, 9.6 in Europe, 13.1 in Southeast Asia, and 14.2 in sub-Saharan Africa [3]. The incidence of neural tube defects is high in Ethiopia, with an estimated pooled prevalence of 63 per 10,000 births [5]. In Addis Ababa, the frequency of NTDs per 10,000 births was 126 at 3 months of pregnancy and 63.4 at 7 months [6].

NTDs include a wide spectrum of embryogenic conditions that are caused by failure of neural tube closure [2, 7]. Spina bifida and anencephaly are the most prevalent types of NTD [2]; however, anencephaly is a lethal type of NTD. A very rare type of NTD known as craniorachischisis is characterized by the presence of both spina bifida and anencephaly in a newborn. Among all NTDs, this is the most severe and fatal form [7]. Despite this, it has been neglected and has only been the subject of limited research until recently [6]. Although low- and middle-income countries have documented reductions in NTDs after the onset of fortification with folic acid, the surveillance efforts for NTDs remain limited in less developed countries [1, 3, 8]. Here, we report two cases of craniorachischisis and one case of discordant twins for anencephaly. Moreover, the relevant works of literature that are necessary to understand and address this unrelenting phenomenon are provided.

## 2. Observations

### 2.1. Case 1: Craniorachischisis Totalis

**Figure 1:**
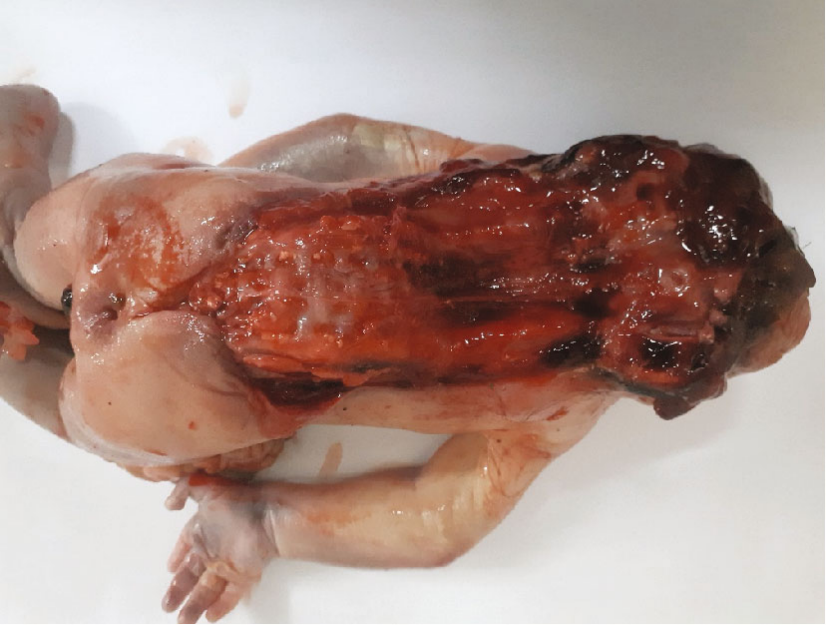
Photograph of a stillborn fetus with craniorachischisis totalis.

A fetus was found dead in a secluded site localized for garbage collection, and police brought it for medicolegal postmortem examination. The body was brought in a cardboard container and wrapped in a black plastic bag. The postmortem examination confirmed that the fetus was a stillborn female and delivered at 36-38 weeks of gestation. On autopsy examination, the scalp and cranial vault were absent, and the brain was malformed and exposed. The forebrain was completely absent, and some rudimentary brain tissue was observed. Specific structures could not be differentiated. The skin over the spine was absent, exposing the spinal cord. Spina bifida was seen with a defect in the continuation of anencephaly and extending from the cervical region to the lower lumbar region. The finding of craniorachischisis totalis was confirmed (Figure 1).

There was no distinguished neck structure; her ears were low set and rotated. The eyes were prominent, closed, and bulging. The nose was broad with a depressed nasal bridge. The mouth was open, the tongue had protruded, and the middle part of the hard palate was split. The umbilical cord was untied, measuring 7 cm from the base to the lacerated end, and it was normal with one vein and two arteries. All other organs were present and normal upon complete postmortem examination. It was not possible to obtain any family or maternal obstetric histories or documentation. Genetic tests were not conducted.

### 2.2. Case 2: Craniorachischisis

**Figure 2:**
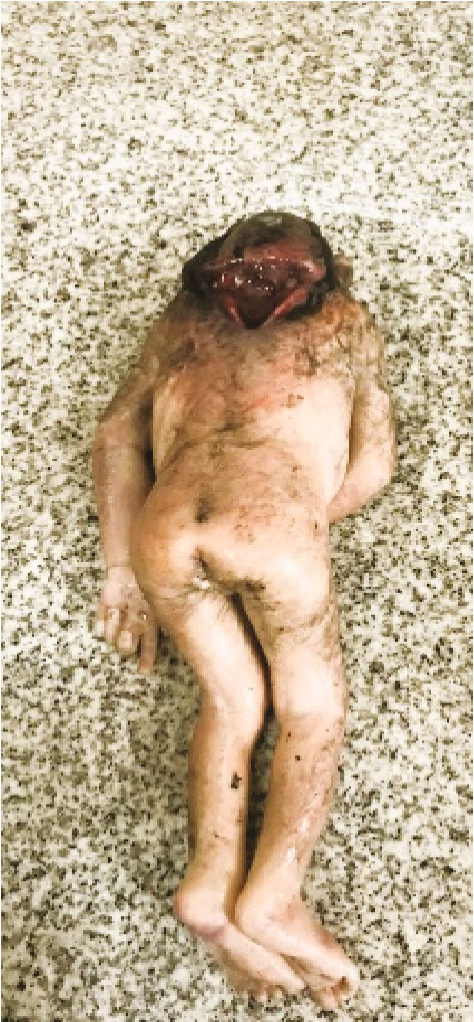
Photograph of a stillborn fetus with craniorachischisis.

A fetus was found dead in a local trash collection site, and police brought it for medicolegal examination. The postmortem examination confirmed that the fetus was a stillborn female and delivered at 38 weeks of gestation. On autopsy examination, the scalp and cranial vault were absent, and the brain was malformed and exposed. The forebrain was completely absent, and some rudimentary brain tissue was observed. Specific structures could not be differentiated. The skin over the spine was absent, exposing the spinal cord. Spina bifida was observed with a defect in the continuation of anencephaly extending up to the lower cervical region. The finding of craniorachischisis was confirmed (Figure 2). There was no distinguished neck structure; her ears were low set and rotated. The eyes were prominent and bulging. The nose was broad with a depressed nasal bridge. The mouth was open, and the middle part of the hard palate was split. The umbilical cord was untied with a lacerated end, and it was normal with one vein and two arteries. No other abnormalities were observed. It was not possible to obtain any family or maternal obstetric histories or documentation. Genetic tests were not conducted.

### 2.3. Case 3: Twin Birth with Female Discordant for Anencephaly

**Figure 3:**
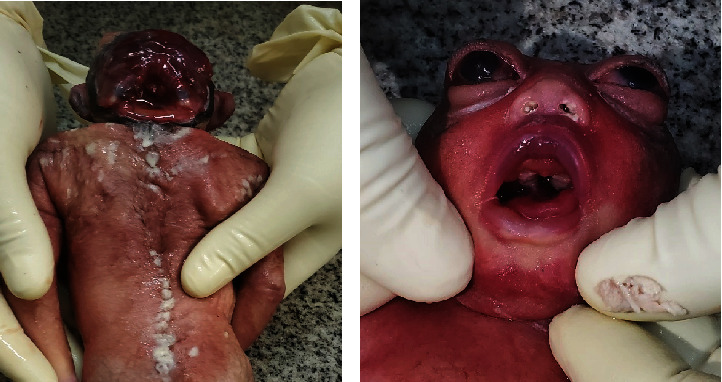
(a) Photograph of a stillborn fetus with anencephaly. (b) Photograph cleft palate and bulging of eyes.

A twin fetus was found dead in a field in the morning, and police brought it for medicolegal postmortem examination. The body was brought in a cardboard container, and upon opening the cardboard box, twin dead fetuses were wrapped with a blood-tinged cloth. The autopsy examination revealed twin fresh stillborn dead bodies with a gestational age of 36 weeks. Twin A was a male stillborn and normal with no abnormalities on complete postmortem examination. The other twin was a female stillborn with anencephaly (Figure 3(a)). The scalp and cranial vault were absent, and the brain was malformed and exposed. Specific brain structures could not be differentiated. The skin covering the spinal cord was intact. The neck was too short; her ears were low set and rotated. The eyes were prominent and bulging. The nose was broad with a depressed nasal bridge. The mouth was open, the tongue had protruded, and the middle part of the hard palate was split (Figure 3(b)). The umbilical cord was untied with a lacerated end, and it was normal with one vein and two arteries. No other abnormalities were observed. It was not possible to obtain any family or maternal obstetric histories or documentation. Genetic tests were not conducted.

## 3. Discussion

Neural tube defects are embryogenic disorders caused by the failure of the formation of neural ectoderm and mesoderm. The neurulation process occurs when the neural plate transforms into a neural tube that forms the primitive nervous system. The neural ectoderm is the precursor to the neural plate. A neural fold forms around the end of the third week of conception as the lateral edge of the neural plate elevates. Eventually, neural folds fuse in the midline and extend cranially and caudally, resulting in the formation of the neural tube. A closed tubular form of the primordial nervous system results from the closure of the cranial neuropore around day 25 and the caudal neuropore around day 28. It is the caudal part of the neural tube that forms the spinal cord and the cranial part that forms the brain. When the neural folds do not close normally during the third and fourth weeks of embryonic development, NTDs occur [9, 10].

Anencephaly occurs when the cranial neuropore does not close. Anencephaly sometimes appears with rachischisis when the spinal cord is partially or completely exposed, and this condition is called craniorachischisis. When the vertebral column and cranial vault do not develop properly, the neural tissue is exposed to significant injury. The defect exposes the neural tissue to destruction by amniotic fluid and additional mechanical trauma to closer structures as the growth of the brain tissue progresses. Then, the exposed neural tissue undergoes secondary degenerative changes that convert it into a mass of rudimentary vascular connective tissue [7, 9, 10]. According to our observations, only rudimentary neural tissue was visible in all three cases, making it difficult to identify specific neural structures. In addition to vertebral and cranial vault defects, bulging of eyes, low-set rotated ears, protruded tongue, broad nose, cleft palate, and small neck were observed in all three cases. Our finding is consistent with other case reports [11, 12].

The rate of NTD varies by geographical location, gender of the fetus, ethnicity, and socioeconomic status of the parents, as well as maternal age. Folate insufficiency in early gestation is a significant risk factor for neural tube defects [13]. Factors such as maternal diabetes, maternal obesity, maternal hyperthermia in early pregnancy, and disruption of the amniotic band during gestation are believed to increase the risk of NTD [2, 6, 7, 11]. Studies have also reported that open NTDs such as craniorachischisis, anencephaly, and spina bifida are more common among females than males [11, 12]. This is supported by our observation that all affected fetuses were females and one male fetus in the twin birth was spared.

Several genes involved in folate metabolism are regarded to play a leading role in the development of NTDs. Folic acid supplementation during the periconceptional period has reduced the risk of NTDs by 72% [7]. Besides, fortification of staple foods with folic acid reduces NTDs [8, 14].

There is extensive evidence showing that low socioeconomic groups usually have poor maternal nutrition, especially folic acid, which increases their risk of NTDs [6, 7, 11]. In all of our observations, the fetuses were found dead outside. Family history, maternal gynecologic, and obstetric history were not available. Therefore, it was difficult to identify the cause. Most likely, these stillborn fetuses belong to a family with low socioeconomic status, and this could also be an unplanned or even extramarital pregnancy. In such cases, there could be no prenatal and antenatal care follow-up, and delivery could also be unattended. Thus, those cases could be from a couple or a single mother with low socioeconomic status and poor maternal nutrition.

Numerous studies have shown that NTDs, such as anencephaly, are more common among twin gestations than among singletons [15–18]. However, the exact reason is still not known. There are severe complications, such as polyhydramnios, premature delivery, and the death of one or both fetuses in twin pregnancies with NTDs, such as anencephaly, as in our third case. Such pregnancies require an individualized management option that considers the potential for severe complications, the layers of the fetal membrane, and the fetal age at diagnosis. Treatment options include selective feticide of the anencephalic fetus, expectant treatment with serial ultrasound monitoring and amnioreduction of polyhydramnios, and abortion of both fetuses [15].

The entry point for proper management is early diagnosis through routine ultrasound examinations. The elevation of alpha-fetoprotein in amniotic fluid and maternal blood also suggests the presence of open NTDs [7, 11]. All infants with anencephaly and craniorachischisis are stillborn or die soon after birth [6]. There is no therapy for such conditions, and they are uniformly lethal. Therefore, if detected early, it is recommended to terminate the pregnancy. For parents with an affected child, genetic counseling and folic acid during the periconceptional period of subsequent pregnancies are recommended prevention measures.

## 4. Surveillance and Prevention of Neural Tube Defects

For prevention and monitoring purposes, robust surveillance data are essential, as congenital anomalies are substantial contributors to childhood mortality. This is especially crucial for congenital abnormalities such as NTD that have proven prevention strategies [1, 2]. In 2010, the World Health Assembly (WHA) urged members to improve birth defect surveillance systems as part of their global efforts to combat birth defects [19]. However, NTD surveillance is still insufficient, especially in African and Southeast Asian regions, underscoring the necessity of strengthening the monitoring system for NTDs [1]. Therefore, we strongly recommend strengthening the surveillance of NTDs and other birth defects using WHA standard reporting tools in developing countries such as Ethiopia.

Folic acid is considered a public health priority when more than 5% of reproductive-age women have a deficiency [20, 21]. An analysis conducted in 2018 found that more than 20% of reproductive-age women have a folic acid deficiency in most low-income countries, while it is less than 5% in high-income countries [22]. Similarly, a recent estimate conducted in Ethiopia found that 20.8% of reproductive-age women had folate deficiency, indicating the public health importance of the problem [21].

The World Health Organization recommends supplementation with folic acid during the periconceptional period, continuing until 3 months after pregnancy. Furthermore, many nations mandate mass folic acid fortification of staple cereals, whereas in Ethiopia, fortification of commonly consumed food vehicles was mainly voluntary [20, 22]. But more recently, in 2022, the Ethiopian Government endorsed the mandatory fortification of edible oil and wheat flour with folic acid [23]. Supplementation with folic acid has been recommended for many years, but studies have shown that many women do not follow it, especially those from low socioeconomic backgrounds [6]. The same is true of a study from Addis Ababa; even if 17% of the study participants received preconception care, only 7.8% received folic acid supplementation [6]. Therefore, preconception care, folic acid supplementation, and obligatory dietary fortification practice should be strengthened in resource-constrained nations such as Ethiopia.

## Data Availability

The data supporting the findings of this study are available within the article. Additional data that supports the findings of this study are available from the corresponding author, upon reasonable request.
